# Analysis of key fishy odorants in crucian carp (*Carassius auratus*) and yellowfin seabream (*Acanthopagrus latus*)

**DOI:** 10.1016/j.fochx.2026.104261

**Published:** 2026-07-29

**Authors:** Yiqi Li, Shu Guo, Chuanbo He, Ruifang Wang, Yuanhong Xie, Hejian Xiong, Ying Ma

**Affiliations:** a Fisheries College of Jimei University, Key Laboratory of Healthy Mariculture for the East China Sea, Ministry of Agriculture and Rural Affairs, Xiamen 361021, China; b College of Ocean Food and Biological Engineering, Jimei University, Xiamen, 361021, China

**Keywords:** Crucian carp (*Carassius auratus*), Yellowfin seabream (*Acanthopagrus latus*), Volatile compounds, Fishy odor, Aroma reconstruction and omission experiment

## Abstract

Fishy odor is a critical factor affecting the sensory quality and consumer acceptance of aquatic products. However, systematic differences in the composition of fishy odor compounds between freshwater and marine fish remain unclear. To address this gap, this study elucidated the composition of fishy odor compounds in freshwater crucian carp (*Carassius auratus*) and marine yellowfin seabream (*Acanthopagrus latus*) through comprehensive analysis of their volatile compounds. The key fishy odorants were identified using headspace solid–phase microextraction (HS-SPME) coupled with gas chromatography–mass spectrometry (GC–MS) for qualitative and quantitative analysis of volatile components, together with the application of odor activity value (OAV), gas chromatography–mass spectrometry-olfactometry (GC–MS-O), as well as aroma recombination and omission experiments. A total of 32 volatile compounds were identified from crucian carp, predominantly aldehydes (283.48 μg/kg), alcohols (144.57 μg/kg), and ketones (116.95 μg/kg). In contrast, 31 volatile compounds were identified from yellowfin seabream, mainly aldehydes (215.17 μg/kg) and hydrocarbons (881.20 μg/kg). Except for hydrocarbons, the concentrations of volatile compounds in crucian carp were higher than those in yellowfin seabream, and 2-methylisoborneol (2-MIB), a known earthy-musty odorant, was detected in crucian carp. Six key fishy odorants in crucian carp included: (E,Z)-2, 6-nonadienal, nonanal, (E,E)-2, 4-decadienal, (E,E)-2, 4-nonadienal, 1-octen-3-ol and hexanal. In yellowfin seabream, four critical odorants were identified: (E,Z)-2, 6-nonadienal, nonanal, (E,E)-2, 4-decadienal and (E,E)-2, 4-nonadienal. The volatile profiles differed markedly between the two species. Compared with the marine yellowfin seabream, the freshwater crucian carp exhibited a more intense odor profile with a more complex composition of fishy odorants.

## Highlights

• Volatile/odor profiles of crucian carp and yellowfin seabream were first established.
• Key fishy odorants in both fish species were identified.
• Odor profile of crucian carp was more intense than yellowfin seabream.
• Crucian carp contained unique earthy-musty odorant 2-MIB.

## Graphical abstract

**Figure:**
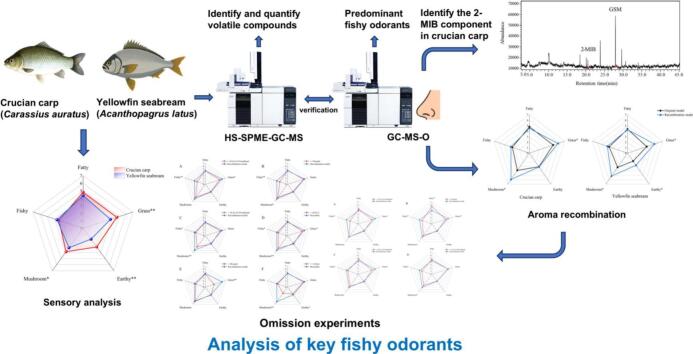

## 1 Introduction

Aquatic products are highly valued for their nutritional benefits, serving as an excellent source of high-quality protein and unsaturated fatty acids, and are widely favored by consumers. However, the characteristic fishy odor of seafood significantly impairs consumer acceptance and diminishes market value. The formation of fishy odor results from the combined action of multiple odorous compounds, primarily originating from three pathways: microbial metabolites in the aquatic environment, lipid oxidation within the fish body, and the decomposition of endogenous trimethylamine N-oxide (TMAO). In freshwater environments, microorganisms such as actinomycetes and cyanobacteria produce geosmin (GSM) and 2-methylisoborneol (2-MIB), which impart earthy-musty odors. These compounds penetrate fish through the gills and skin, contributing to the typical off-flavors in freshwater species (Robertson et al., 2006). Additionally, fish are rich in unsaturated fatty acids (UFAs), and the oxidative degradation of UFAs generates low-molecular-weight volatile aldehydes, ketones, and alcohols, which are main contributors to fishy odor (Yarnpakdee et al., 2012). Furthermore, TMAO is widely present in various aquatic products, particularly in sea foods. The decomposition of TMAO releases trimethylamine (TMA), dimethylamine, and methylamine, which are the main sources of the unpleasant fishy odor in spoiled aquatic products (Dai et al., 2024).

Crucian carp (*Carassius auratus*) and yellowfin seabream (*Acanthopagrus latus*) are important aquaculture fish species in China. In 2023, their aquaculture production reached 2,840,261 tons and 147,314 tons, respectively (Bureau of Fisheries and Fishery Administration et al., 2024). They are also the primary raw materials for making traditional and processed fish soup in both inland and coastal regions of China. Fish soup is a classic dish in Chinese culinary culture, highly valued for its nutritional and restorative properties. It is widely consumed and deeply favored in household diets. Additionally, it is a traditional practice in some Chinese communities to prepare lightly salted fish soup for postpartum women to promote lactation. Although the nutritional value of fish soup is widely recognized by consumers, its relatively pronounced fishy odor—due to the minimal use of seasonings during processing—makes it less acceptable to younger consumers. Investigating the compounds responsible for the fishy odors and developing effective deodorization techniques are common challenges in improving consumer acceptance of aquatic processed products such as fish soup. Systematic analysis of the key fishy odor compounds in the raw materials, crucian carp and yellowfin seabream, is a prerequisite and scientific foundation for the precise regulation of flavor in their soup products. Only by establishing a “baseline odor chemistry profile” for the raw materials can we further elucidate the evolution of flavor during processing and design targeted deodorization strategies.

Although research on fish volatile compounds has been extensive, current studies have largely focused on characterizing the aroma profiles of individual fish species or on merely identifying potential fishy odorants based on odor activity value (OAV) and gas chromatography-olfactometry (GC-O) (Geng et al., 2022; Liu et al., 2021). A significant gap remains: no study has yet conducted a direct, systematic comparison of freshwater crucian carp and marine yellowfin seabream using a “molecular sensory science” approach (Wang et al., 2024). Such a comparison would not only identify the key fishy odorants in each species but could also, by revealing differences in their volatile compound profiles, reflect to a certain extent the potential influence of distinct aquatic habitats on the formation of volatile metabolites in fish. This would provide a chemical perspective for understanding the ecological basis of odor formation. Furthermore, existing methods predominantly rely on instrumental analysis and fail to directly connect sensory perception with the verification of causal relationships.

This study follows the molecular sensory science approach of Schieberle's team, which closely integrates instrumental analysis with human sensory evaluation (Wang et al., 2024). Crucian carp (a freshwater fish) and yellowfin seabream (a marine fish) were selected as representative raw materials for traditional Chinese fish soup. The overall odor profiles were characterized through sensory evaluation, and volatile compounds were identified and quantified using gas chromatography–mass spectrometry (GC–MS). By integrating gas chromatography–mass spectrometry-olfactometry (GC–MS-O) and OAV analysis, fishy odor-associated compounds in the fish were identified. Aroma reconstruction and omission tests were further conducted to confirm the key fishy odorants. The findings provide a theoretical foundation for future research on storage and processing improvements of these fish species.

## 2 Materials and methods

### 2.1 Experimental fish and chemical reagents

Live crucian carp and yellowfin seabream, with an average body weight of 0.5–1 kg per fish, were purchased from Jianong Market in Xiamen, China. The fish were immediately transported to the laboratory in aerated tanks containing fresh water (for crucian carp) or seawater (for yellowfin seabream) to minimize stress. Upon arrival, the fish were immediately euthanized by percussive stunning to minimize suffering. After removing gills and viscera, the fish were rinsed with distilled water, segmented, and stored at 4 °C for further use.

Standard volatile compounds (see Table S1 for full details) were obtained from Sigma-Aldrich (St. Louis, MO, USA). All reagents were of analytical or chromatography grade.

### 2.2 Sensory evaluation

Sensory evaluation was conducted according to ISO 8586:2012, following the method described by Ni et al. (2021). Through three preparatory sessions (3 h each), grassy, fatty, mushroom, fishy, and earthy odors were unanimously selected as the sensory descriptors. Twelve assessors (6 males and 6 females, aged 20–30) were first familiarized with the above odor characteristics and intensities using reference solutions: hexanal (grassy), nonanal (fatty), 1-octen-3-ol (mushroom), trimethylamine (fishy), and geosmin (earthy) (An et al., 2020), until they could accurately describe and discriminate each attribute. Formal evaluation was performed in a clean, controlled environment (22 ± 2 °C, 55 ± 2% relative humidity) using a 0–10 point intensity scale (0 = not perceived, 10 = extremely strong). Each sample was assessed in triplicate by all panelists on different days, and the results were averaged for data analysis.

### 2.3 Extraction of volatiles by HS-SPME

Fifteen grams of fish muscle were weighed and placed into a 60 mL headspace vial, followed by the addition of 15 mL of saturated sodium chloride solution and 5 μL of the internal standard cyclohexanone (1 mg/mL). The mixture was equilibrated at 60 °C for 10 min in a water bath. A solid-phase microextraction (SPME) fiber assembly (57330-U, Supelco, Bellefonte, PA, USA) was inserted into the headspace to adsorb volatile compounds for 40 min. Upon completion of extraction, the SPME fiber (65 μm DVB/CAR/PDMS, Supelco, USA) was promptly removed and introduced into the injection port of a GC–MS system (QP-2010Plus, Shimadzu, Kyoto, Japan) for thermal desorption over 3 min.

### 2.4 Qualitative and quantitative analysis of volatiles using GC–MS

Volatile compounds in fish muscle were qualitatively and quantitatively analyzed using a QP-2010Plus GC–MS system (Shimadzu, Kyoto, Japan) equipped with an Rtx-5MS capillary column (30 m × 0.25 mm × 0.25 μm; Restek Corporation, Bellefonte, PA, USA). High-purity helium (99.999%) was used as the carrier gas at a constant flow rate of 1 mL/min in splitless injection mode. The oven temperature was programmed as follows: initial temperature of 40 °C held for 2 min, increased to 160 °C at a rate of 4 °C/min, then raised to 250 °C at 10 °C/min, and finally held at 250 °C for 5 min. Mass spectrometry (MS) analysis was operated in electron impact (EI) mode with an ionization energy of 70 eV. The ion source and interface temperatures were set at 230 °C and 250 °C, respectively. The mass scanning range was set from 35 to 350 *m*/*z*.

Compounds with available reference standards were identified by matching their RI and characteristic mass spectral fragments with those of the authentic standards. The remaining components were tentatively identified by comparing their mass spectra with those in the NIST11, NIST11s, and FFNSC1.3 mass spectral databases, with only those compounds exhibiting a similarity greater than 80% being selected for qualitative analysis. Additionally, a homologous series of alkanes mixtures (C8-C20) was analyzed under the same chromatographic conditions to calculate the RI of the volatile compounds for further confirmation.

Quantitative analysis was performed using selected ion monitoring (SIM) mode and carried out via an internal standard-calibrated external standard method. A series of standard solutions at different concentrations were prepared by gradient dilution to establish calibration curves (Table S2). Compounds with established calibration curves were quantified based on their respective curves. For volatile compounds without a calibration curve, the relative contents (μg/kg) were estimated semi-quantitatively using the following formula: (peak area of analyte / peak area of internal standard) × concentration of internal standard.

### 2.5 OAV calculation

OAV is used to evaluate the contribution of an individual volatile compound to the overall fishy odor. It is calculated as follows: OAV = C/T, where C represents the concentration of the volatile compound in the sample (μg/kg), and T denotes the sensory threshold (i.e., the minimum perceivable concentration, μg/kg) for that compound. Threshold values were obtained from previously published literature.

### 2.6 GC–MS-O analysis of odor characteristics

The GC–MS-O analysis was performed using an Rtx-5MS capillary column (60 m × 0.25 mm × 0.25 μm; Restek Corporation, Bellefonte, PA, USA). The GC–MS conditions were the same as described in Section 2.4. After solid-phase microextraction, the samples were subjected to chromatographic separation and were directed via a splitter at a split ratio of 16:9 to the mass spectrometer and the olfactory detection port (ODP-2, Gerstel, Germany), respectively. The injection was performed in splitless mode. The temperature of the sniffing port was maintained at 250 °C, and the humidified airflow rate was set at 50 mL/min. Compound identification followed the same criteria as in Section 2.4.

The odor evaluation was performed by five trained assessors (3 males and 2 females, aged 20–30 years) following the ISO 8586:2012 standard. The odor perception intensity was recorded based on the method described by Ni et al. (2021). The intensity levels were categorized as follows: 1 (extremely weak), 2 (weak), 3 (moderate), 4 (strong), and 5 (extremely strong). Each assessor independently conducted three replicate evaluations. A compound was deemed a valid odor-active substance only if it was perceived by at least two assessors in two or more replicates, with consistent description of its odor attributes.

### 2.7 Aroma reconstruction and omission test

Aroma reconstruction: An aroma recombinant model was prepared in 0.2 mol/L phosphate buffer (pH 6.8) by incorporating the key odorants (OAV ≥ 1) identified in the fish samples. Sensory evaluation of the recombinant models for both fish species was conducted following the method described in Section 1.2. The similarity between the aroma profile of the recombinant model and that of the original sample was evaluated using the cosine similarity method. A cosθ value closer to 1 indicates a higher similarity between the model and the actual sample (Xie et al., 2017). The cosine θ value was calculated as follows:

$$\cos\theta =\frac{\sum_{k=1}^{n}X_{\mathrm{ik}}∙X_{\mathrm{rk}}}{\sqrt{\left(\sum_{k=1}^{n}X_{\mathrm{ik}}^{2}\right)\left(\sum_{k=1}^{n}X_{\mathrm{rk}}^{2}\right)}}$$

In this context, *X*_*ik*_ and *X*_*rk*_ are the sensory intensity scores of attribute *k* (where *k* = 1,2, …, n) in the original sample *i* and the reconstruction model *r*, respectively. The cosine similarity was calculated using Excel with the following function: “=SUMPRODUCT (Xrk, Xik) / SQRT (SUMSQ (Xik) * SUMSQ (Xrk))”.

Omission experiment: Following the method described by Xue et al. (2019), a triangle test was employed to evaluate the contribution of components associated with fishy odor (GC-O sniffing intensity ≥2 and OAV ≥ 10). Individual components were omitted sequentially from the reconstruction model to analyze the significance of their absence on the overall aroma profile. For components that exhibited a significant impact, further sensory evaluations were conducted using the same methods and data processing approach as those applied in the reconstruction experiment.

### 2.8 Data analysis

Data are presented as mean ± SD. Statistical analysis of significant differences and calculation of means and standard errors were performed using Excel 2010 (Microsoft Corp., USA) and SPSS (IBM Corp., USA). Radar charts were generated using Origin 2022 software (OriginLab Corporation, USA). Heatmaps were constructed using Hemi 1.0.3.7 software (CUCKOO Workgroup, China).

## 3 Results

### 3.1 Sensory evaluation of fresh fish

**Fig. 1:**
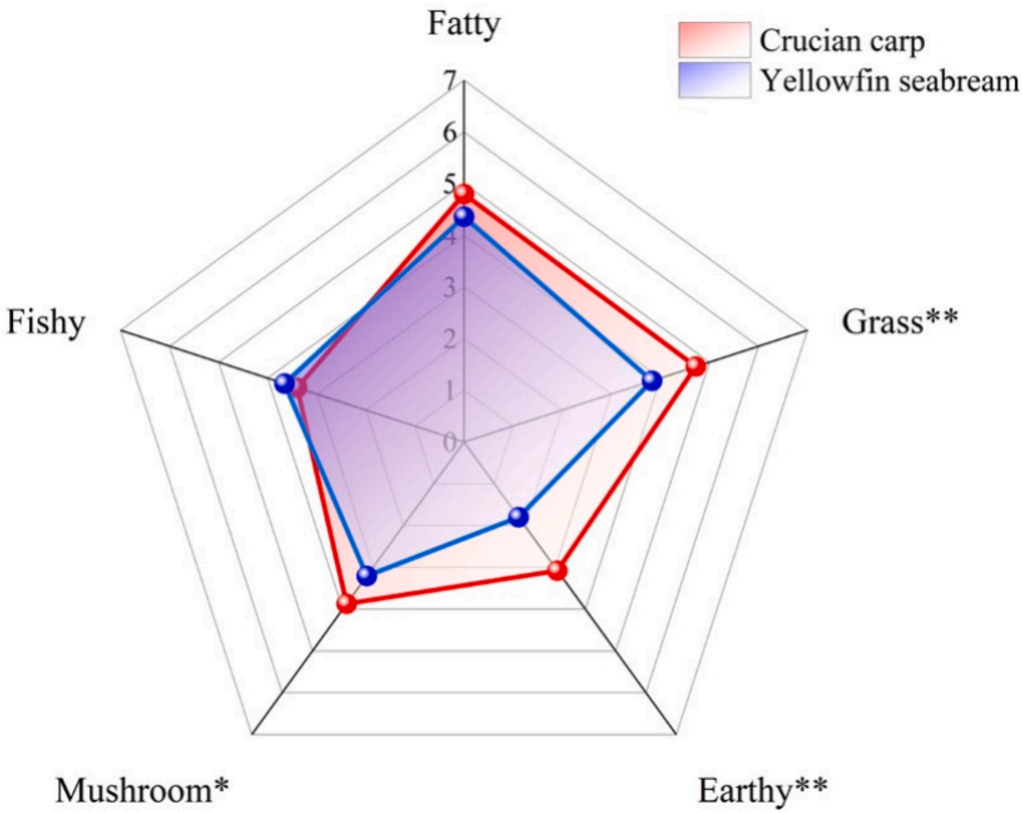
The odor profile of fresh crucian carp and yellowfin seabream (* and ** indicate significant difference at *P* < 0.05and *P* < 0.01, respectively).

Sensory evaluation revealed distinct aroma profiles for crucian carp and yellowfin seabream, as illustrated in the radar chart (Fig. 1). Key odor descriptors included fishy, fatty, grassy, mushroom, and earthy notes. For crucian carp, the intensity scores for fatty, grassy, earthy, mushroom, and fishy odors were 4.8, 4.73, 3.08, 3.87, and 3.4, respectively; the corresponding scores for yellowfin seabream were 4.36, 3.83, 1.8, 3.21, and 3.6. The sensory evaluation revealed that crucian carp was rated higher than yellowfin seabream across all odor attributes. While the differences in fishy and fatty notes were not statistically significant, the intensities of earthy, grassy, and mushroom odors were significantly stronger in crucian carp (*P* < 0.01 or *P* < 0.05). Similarly, Yueqi et al. (2024) reported significantly higher “fresh fish”, “grassy”, and “earthy” notes in freshwater silver carp surimi than in marine fish products.

### 3.2 Qualitative, quantitative, and OAV analysis of volatile components

The qualitative analysis results of volatile compounds in crucian carp and yellowfin seabream are shown in Table 1, and the quantitative results and OAVs are presented in Table 2. A total of 36 volatile compounds were identified, including 16 aldehydes, 8 alcohols, 6 hydrocarbons, 2 ketones, 2 aromatic compounds, and 2 other compounds. Crucian carp exhibited notably higher concentrations of aldehydes, alcohols, and ketones (283.48 μg/kg, 144.57 μg/kg, and 116.95 μg/kg, respectively) compared to yellowfin seabream (215.17 μg/kg, 69.47 μg/kg, and 28.32 μg/kg, respectively). In contrast, the concentrations of hydrocarbons and other compounds were lower in crucian carp than those in yellowfin seabream. The OAV reflects the odor intensity of a volatile compound in the sample. In both fish species, volatile compounds with OAV ≥ 1 belonged to aldehydes, ketones, and alcohols. A total of 10 compounds showed OAV ≥ 10, among which 7 were associated with a fishy odor: (E,Z)-2, 6-nonadienal, (E,E)-2, 4-nonadienal, 1-octen-3-ol, (E,E)-2, 4-decadienal, hexanal, nonanal, and decanal. Their corresponding OAVs in crucian carp were 267, 85, 43, 42, 41, 21, and 14, while those in yellowfin seabream were 571, 85, 38, 42, 22, 13, and 15, respectively.

**Table 1:** Identified volatile components in crucian carp and yellowfin seabream.

Components	RI_1_	RI_2_	Identification	CI	PubChem CID	Crucian carp	Yellowfin seabream
Hexanal	802	802	Std MS RI	44,56,72	6184	Y	Y
Heptanal	900	899	Std MS RI	44,70	8130	N	Y
E-2-Heptenaldehyde	955	956	MS RI	41,55,83	5,283,316	Y	Y
Octanal	1003	1003	Std MS RI	43,56,84	454	Y	Y
(E,E)-2,4-Heptadienal	1011	1011	Std MS RI	81,53,110	5,283,321	Y	Y
E-2-Octenal	1057	1056	Std MS RI	41,55,70	5,283,324	Y	Y
(Z,Z)-3, 6-Nonadienal	1094	1097	MS RI	67,41,55	5,352,808	N	Y
Nonanal	1104	1103	Std MS RI	57,41,70	31,289	Y	Y
(E,Z)-2, 6-Nonadienal	1150	1151	Std MS RI	41,70,53	643,731	Y	Y
E-2-Nonenal	1158	1158	Std MS RI	41,55,70	5,283,335	Y	Y
Decanal	1204	1204	Std MS RI	43,57,70	8175	Y	Y
(E,E)-2, 4-Decadienal	1212	1212	Std MS RI	81,41	5,283,339	Y	Y
E-2-Decenal	1259	1259	Std MS RI	41,55	5,283,345	Y	Y
(E,E)-2, 4-Decadienal	1314	1314	Std MS RI	81,41,55	5,283,349	Y	Y
(E,E)-2,4-Dodecadienal	1290	1295	Std MS RI	81,41,55	5,367,530	Y	N
Dodecanal	1405	1402	Std MS RI	57,82	8194	Y	Y
2,3-Butanediol	<800	782	MS RI	45,57,75	262	Y	N
Hexanol	866	865	Std MS RI	56,43,69	8103	Y	N
Heptanol	971	971	Std MS RI	57,70,41	8129	Y	Y
1-Octen-3-ol	981	980	Std MS RI	57,43,72	18,827	Y	Y
2-Ethylhexanol	1029	1029	Std MS RI	57,41,70	7720	Y	Y
(E)-2-Octen-1-ol	1067	1067	Std MS RI	57,41	5,318,599	Y	N
Octanol	1071	1071	Std MS RI	56,41,70	957	Y	Y
Nonanol	1171	1171	Std MS RI	56,41,70	8914	Y	Y
2,3-Octanedione	985	984	MS RI	43,71,99	11,449	Y	Y
3,5-Octadien-2-one	1092	1093	Std MS RI	95,81,43	5,352,876	N	Y
Dodecane	1200	1200	Std MS RI	57,43,71	8182	Y	Y
Tridecane	1299	1300	Std MS RI	41,55	12,388	Y	Y
Tetradecane	1397	1400	Std MS RI	57,43,71	12,389	Y	Y
Pentadecane	1497	1500	Std MS RI	57,43,71	12,391	Y	Y
Hexadecane	1596	1600	Std MS RI	57,43,71	11,006	Y	Y
8-Heptadecene	1665	1670	MS RI	55,69,41	5,364,555	Y	Y
Toluene	<800	785	MS RI	91,65,39	1140	N	Y
Naphthalene	1178	1174	Std MS RI	128,102,51	931	Y	Y
Caryophyllene	1412	1414	Std MS RI	93,41,133	5,281,515	Y	N
Dibutyl phthalate	1849	1865	Std MS RI	149,57,41	6782	Y	Y

Note: “RI_1”_ refers to the calculated retention index; “RI_2”_ corresponds to the retention index provided in the NIST Chemistry WebBook; “Std” indicates verification using reference standards; “MS” denotes identification by mass spectrometry databases (NIST08, NIST08s, FFNSC1.3); “CI” refers to characteristic ion fragments, and PubChem CID is the identifier for the compound in the NCBI database; “Y” indicates detected, while “N” indicates not detected.

**Table 2:** Quantitative analysis, odor thresholds, and OAVs of odor compounds in crucian carp and yellowfin seabream.

Compounds	Threshold (μg/kg)	Concentration (μg/kg)	OAV			Odor description
		Crucian carp	Yellowfin seabream	Crucian carp	Yellowfin seabream	
Hexanal	4.5 (Guo et al., 2019; Ma et al., 2020)	185.66 ± 26.76^a^	100.88 ± 4.27^b^	41	22	Grassy, Fishy
Heptanal	2.8 (Zhang et al., 2023)	ND	20.53 ± 0.64	ND	7	Fatty, Fishy
E-2-Heptenaldehyde	13 (Zhou et al., 2016)	9.76 ± 2.74^a^	7.92 ± 0.12^b^	<1	<1	Fruity, Fatty
Octanal	3.4 (An et al., 2020)	27.01 ± 0.18^a^	27.44 ± 0.15^a^	8	8	Grassy
(E,E)-2, 4-Heptadienal	15.4 (Zhou et al., 2016)	14.70 ± 0.04^a^	10.63 ± 0.11^b^	1	<1	Fatty, Fishy, Grassy
E-2-Octenal	3.0 (An et al., 2020; Zhou et al., 2016)	2.45 ± 0.21^a^	1.70 ± 0.07^b^	<1	<1	Fatty
(Z,Z)-3, 6-Nonadienal	0.68 (Van Gemert, 2011)	ND	10.64 ± 1.65	ND	16	Cucumber, Fatty
Nonanal	1.0 (Caprino et al., 2008)	20.55 ± 3.33^a^	13.40 ± 1.01^b^	21	13	Fatty, Fishy, Grassy
(E,Z)-2, 6-Nonadienal	0.0045 (An et al., 2020; Mall & Schieberle, 2017)	1.20 ± 0.01^b^	2.57 ± 0.04^a^	267	571	Cucumber, Fishy, Grassy
E-2-Nonenal	0.1 (Caprino et al., 2008)	2.02 ± 0.05^a^	1.97 ± 0.01^a^	20	20	Fatty
Decanal	0.1 (Gu et al., 2013; Zhuang et al., 2016)	1.41 ± 0.05^b^	1.47 ± 0.05^a^	14	15	Fatty, Fishy
(E,E)-2, 4-Nonadienal	0.046 (An et al., 2020)	3.90 ± 0.01^a^	3.89 ± 0.01^a^	85	85	Irritant, Fishy
E-2-Decenal	0.3 (Van Gemert, 2011)	6.12 ± 0.66^a^	5.74 ± 0.37^a^	20	19	Nutty, Fatty
(E,E)-2, 4-Decadienal	0.07 (An et al., 2020)	2.97 ± 0.01^a^	2.94 ± 0.01^b^	42	42	Cucumber, Fishy, Grassy
(E,E)-2, 4-Dodecadienal	0.5	4.10 ± 0.29	ND	8	ND	Fatty, Insect
Dodecanal	0.53 (Van Gemert, 2011)	1.63 ± 0.23^b^	3.45 ± 0.64^a^	3	7	Fatty, Pine scent
2,3-Butanediol	95.1 (Giri et al., 2010)	28.10 ± 8.25	ND	<1	ND	Floral, Fruity
Hexanol	2500 (Ma et al., 2020; Wang et al., 2018)	37.03 ± 2.21	ND	<1	ND	Grassy
Heptanol	5.4 (Giri et al., 2010)	17.78 ± 0.02^a^	17.81 ± 0.14^a^	3	3	Fresh fragrance
1-Octen-3-ol	1.2 (An et al., 2020)	51.27 ± 1.51^a^	45.41 ± 0.28^b^	43	38	Mushroom, Earthy, fishy
2-Ethylhexanol	2548.2 (Zhang, Wang & Shi, 2019)	1.56 ± 0.30^b^	1.83 ± 0.08^a^	<1	<1	Raw fish, Earthy
(E)-2-Octen-1-ol	20 (Van Gemert, 2011)	4.06 ± 0.21	ND	<1	ND	Mushroom, Metallic
Octanol	3.4 (An et al., 2020; Mall & Schieberle, 2017)	2.01 ± 0.07^a^	1.90 ± 0.02^b^	<1	<1	Oily, Grassy
Nonanol	1.0 (Frank et al., 2009)	2.76 ± 0.10^a^	2.52 ± 0.01^b^	3	2	Grassy, Fatty
2,3-Octanedione	12 (Zhang et al., 2023)	116.95 ± 14.00^a^	25.81 ± 5.70^b^	9	2	Buttery
3,5-Octadien-2-one	0.87 (Zhang, Gao, et al., 2022)	ND	2.51 ± 0.32	ND	3	Creamy, Fruity
Dodecane	2040 (Gu et al., 2013)	4.85 ± 0.69^b^	12.92 ± 1.94^a^	<1	<1	Irritant
Tridecane	2140 (Gu et al., 2013)	11.39 ± 4.42^b^	37.39 ± 4.08^a^	<1	<1	Irritant
Tetradecane	1400 (Gu et al., 2013)	13.69 ± 4.93^b^	62.42 ± 6.67^a^	<1	<1	Irritant
Pentadecane	1500 (Gu et al., 2013)	34.83 ± 3.10^b^	715.99 ± 78.58^a^	<1	<1	Irritant
Hexadecane	ND	38.66 ± 6.54^a^	30.57 ± 4.80^b^	ND	ND	Irritant
8-Heptadecene	ND	3.63 ± 0.32^b^	21.91 ± 3.48^a^	ND	ND	Earthy
Toluene	527 (Zhang, Yang, et al., 2022)	ND	68.52 ± 9.58	ND	<1	Similar to benzene
Naphthalene	60 (Gu et al., 2013)	4.67 ± 1.21^a^	5.06 ± 1.04^a^	<1	<1	Camphor ball
Caryophyllene	150(Van Gemert, 2011)	4.59 ± 0.45	ND	<1	ND	Earthy, Fresh fragrance
Dibutyl phthalate	ND	6.49 ± 0.65^b^	45.35 ± 8.17^a^	ND	ND	ND

Note: Except for the threshold of (E,E)-2,4-dodecadienal, which was determined in this study by preparation and measurement, the thresholds and odor descriptions of all the other compounds were referenced from the literature listed in the table and the following publications (Guo et al., 2024; Wongsa et al., 2025). “ND” indicates that the threshold for the volatile component is unknown, below the detection limit, or that its odor description has not been clearly defined. Lowercase letters “a” and “b” denote a significant difference (*P* < 0.05) in the same volatile component between the two fish species.

### 3.3 GC–MS-O analysis and identification of 2-MIB

The identification and olfactory results from GC–MS-O are presented in Table 3. A total of 25 volatile compounds were detected in crucian carp and yellowfin seabream, including 12 aldehydes, 1 alcohol, 1 ketone, 1 hydrocarbon, 1 ester, and 9 unidentified components.

**Table 3:** GC–MS-O analysis of odor–active compounds in crucian carp and yellowfin seabream.

Components	Odor description	Retention time (min)	Odor intensity	
			Crucian carp	Yellowfin seabream
Hexanal	Grassy	7.14	2	1.2
Unknown 1	Cloying, Fatty, Grassy	9.61	ND	1.03
Unknown 2	Fatty, Meaty aroma	10.43	2.63	2.43
Unknown 3	Potato chips, Grilled meat, Fatty	10.79	3.02	2.23
E-2-Heptenaldehyde	Meaty, Milky	11.32	ND	2.57
Unknown 4	Grassy, Fishy	11.5	1.25	ND
1-Octen-3-ol	Mushroom, Earthy, Fishy	13.45	3.12	2.05
E-2-Octenal	Fatty, Cloying, Milky	16.47	2.64	1.5
Unknown 5	Milky	17.45	2.5	ND
3,5-Octadien-2-one	Cloying, Milky	17.85	2.18	2.08
Nonanal	Grassy, Fishy, Pungent	19.68	2.33	2.2
(E,Z)-2, 6- Nonadienal	Cucumber, Grassy, Pungent	19.88	3.77	3.82
Unknown 6	Geosmin	20.18	1.88	ND
(E)-2-Nonenal	Pungent, Peculiar	20.74	1.33	1.9
Decanal	Fatty, Fishy	21.36	1.17	1.67
(E,E)-2, 4-Nonadienal	Insect, Fatty, Fishy	22.14	2.75	2.9
Unknown 7	Insect, Fishy, Grassy	22.95	1.27	1.9
E-2-Decenal	Unpleasant taste	24.15	1.33	1.1
(E,E)-2,4-Dodecadienal	Fatty, Fishy, Grassy	24.88	2.75	ND
Unknown 8	Fatty, Fishy, Earthy	25.37	2.53	1.5
(E,E)-2, 4-Decadienal	Insect, Fishy, Grassy	25.64	3.46	3.23
Dodecanal	Fatty	26.71	ND	1.93
Unknown 9	Earthy, Leather, Plastic	27.69	2.85	1.83
8-Heptadecene	Grassy, Earthy	30.9	1.1	1.63
Dibutyl phthalate	Fatty	34.32	1.53	2.18

Note: “Odor description” refers to the olfactory note perceived through the sniffing port; “Odor intensity” indicates the intensity perceived through the same port; “ND” indicates not detected.

Among the identified substances that were directly described as contributing to fishy odor, six compounds were recognized: 1-octen-3-ol, nonanal, decanal, (E,E)-2, 4-nonadienal, (E,E)-2, 4-dodecadienal, and (E,E)-2, 4-decadienal. Notably, (E,E)-2, 4-dodecadienal was only detected in crucian carp, with an intensity rating of 2.75. The other five compounds have been frequently reported in literature as representative contributors to fishy odor (An et al., 2020; Zhuang et al., 2016).

(E,Z)-2, 6-Nonadienal is frequently reported in the literature as a fishy odor compound (An et al., 2020). Although it was not directly described as having a fishy smell in this study, its odor intensity was the highest in both crucian carp and yellowfin seabream (with scores of 3.77 and 3.82, respectively). Most assessors described it as having a strong, pungent odor, indicating that this compound may impart undesirable odors to the fish and potentially intensify the fishy odor significantly. In this study, hexanal showed relatively low odor intensity in both crucian carp and yellowfin seabream (with scores of 2.00 and 1.20, respectively), and was described as “grassy” rather than “fishy”. However, several studies have identified hexanal as one of the representative compounds of fishy odor (Guo et al., 2024; Liu et al., 2024; Zhang et al., 2023). This discrepancy may be attributed to the low concentration of hexanal in the fresh fish samples used in this study, as the odor characteristics of hexanal are concentration-dependent: it presents a grassy note at low concentrations (Duan et al., 2021), while at higher concentrations or in the presence of other odorants, it can exhibit fishy odor (Guo et al., 2019; Wang, Liu, et al., 2023). Additionally, seven compounds, including E-2-heptenal, were primarily described in this study as having meaty or creamy aromas, and most literature does not classify them as contributors to fishy odors.

A total of nine unknown compounds were detected in the experiment. Three of them (unknowns 4, 7, and 8) were described as having a fishy odor through olfactory assessment. However, their odor intensities were weak. Unknown compound 6 was only perceived in crucian carp, exhibiting typical earthy-musty odor characteristics, but its intensity was low (score: 1.88, Table 3), and no distinct chromatographic peak was observed, likely due to its concentration being below the detection limit of the instrument. Since GSM and 2-MIB are key earthy-musty odorants in freshwater fish (Liu et al., 2021), we hypothesized that unknown 6 could be one of them.

**Fig. 2:**
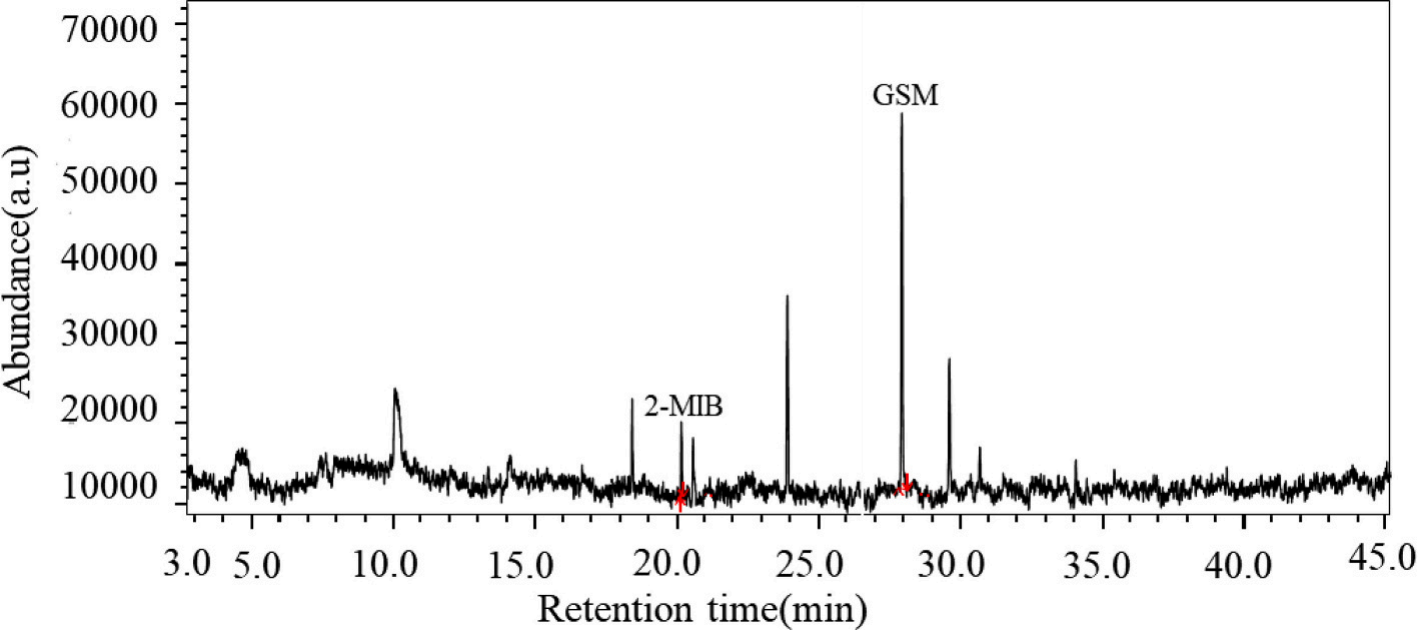
Total ion diagrams of 2-MIB and GSM.

To further validate unknown compound 6, a targeted GC–MS-O analysis was conducted using standard samples of 2-MIB and GSM, with the total ion chromatograms presented in Fig. 2. The retention time (20.18 min) and calculated RI (1159) of 2-MIB matched those of unknown compound 6, while GSM eluted later (28.04 min, RI: 1417), consistent with literature values (San-Juan et al., 2010; Suriyaphan et al., 2001). In the olfactory evaluation, the odor of the 2-MIB standard was characterized as earthy and musty, aligning with the perceived characteristics of unknown 6. Furthermore, a calibration experiment confirmed that 2-MIB could be reliably detected by GC–MS-O only at concentrations ≥60 ng/L, explaining why this compound in our sample was olfactorily perceptible but not initially visible in the chromatogram. Therefore, unknown compound 6 was identified as 2-MIB.

**Fig. 3:**
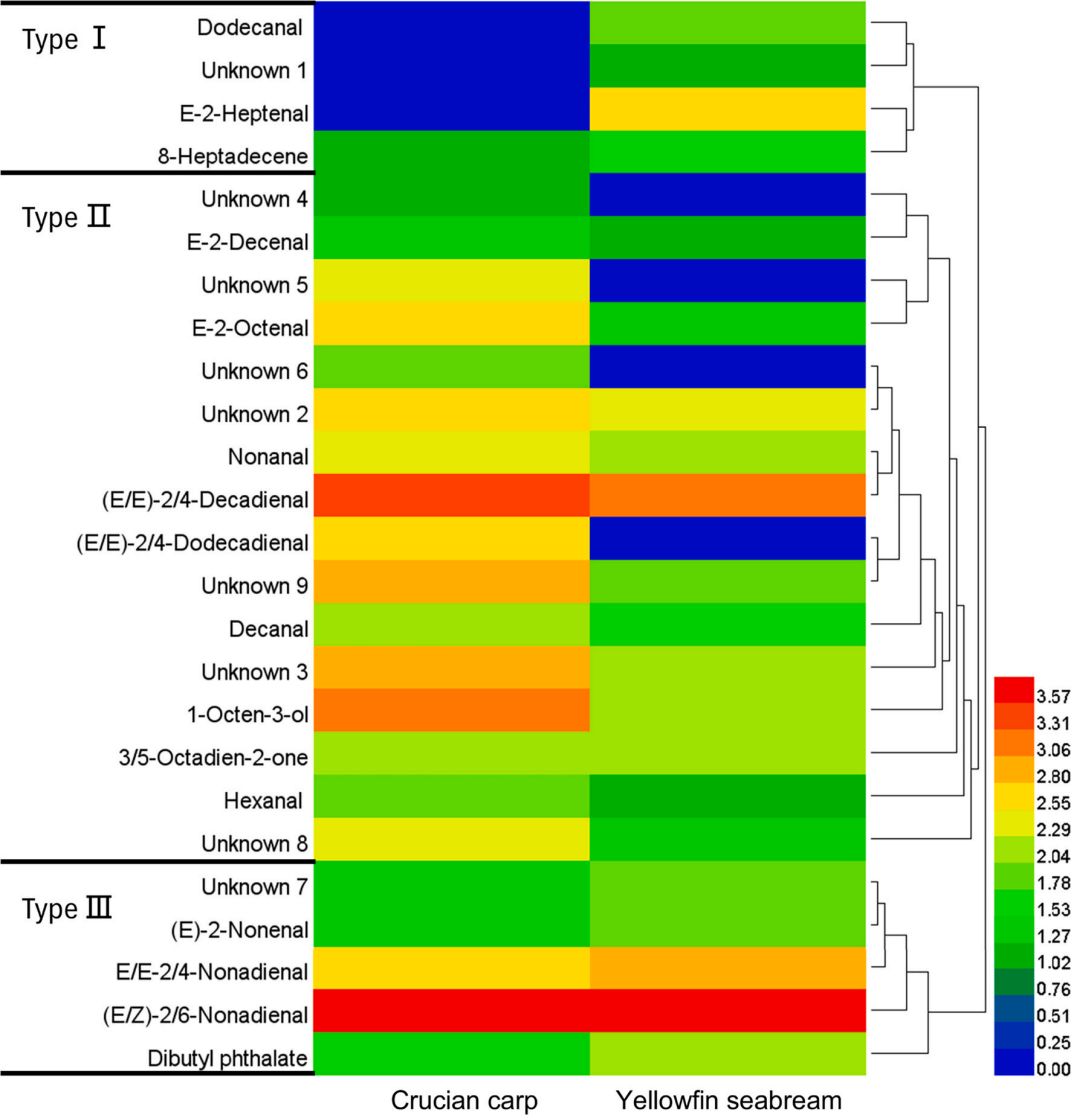
GC–MS-O heat map analysis of volatile compounds in crucian carp and yellowfin seabream.

To investigate the differences in overall odor profiles between crucian carp and yellowfin seabream, a heatmap was constructed based on the aroma intensities of volatile compounds (Fig. 3). As shown in the figure, the odor-active compounds in the two fish species can be categorized into three groups. The first group includes four compounds with relatively weak odor intensities: dodecanal, unknown compound 1, E-2-heptenal, and 8-heptadecene, which are characterized by higher odor intensities in yellowfin seabream than in crucian carp and primarily presenting fatty and grassy notes. The second group comprises 16 compounds, including nonanal, E-2-decenal, and hexanal, which demonstrated higher odor intensities in crucian carp than in yellowfin seabream. These compounds are mainly associated with fishy, mushroom, grassy, and earthy odors, consistent with sensory evaluations results. Notably, this group includes 2-MIB, a compound known for its typical earthy-musty odor. The third group consists of five compounds, among which two compounds with higher odor intensities, namely (E,E)-2, 4-nonadienal and (E,Z)-2, 6-nonadienal, exhibited similar odor intensities in both yellowfin seabream and crucian carp. The results of the heatmap analysis also indicated that the overall odor intensity of volatile compounds in crucian carp was higher than that in yellowfin seabream, which was consistent with the findings of sensory evaluation.

In summary, a total of eight fishy odor-associated compounds were identified in crucian carp and yellowfin seabream through GC–MS-O analysis. These compounds include 1-octen-3-ol, nonanal, decanal, (E,E)-2,4-nonadienal, (E,E)-2, 4-dodecadienal, (E,E)-2, 4-decadienal, (E,Z)-2, 6-nonadienal and hexanal. Notably, 2-MIB, a representative compound responsible for earthy odors, was identified specifically in crucian carp.

### 3.4 Aroma recombination analysis

**Fig. 4:**
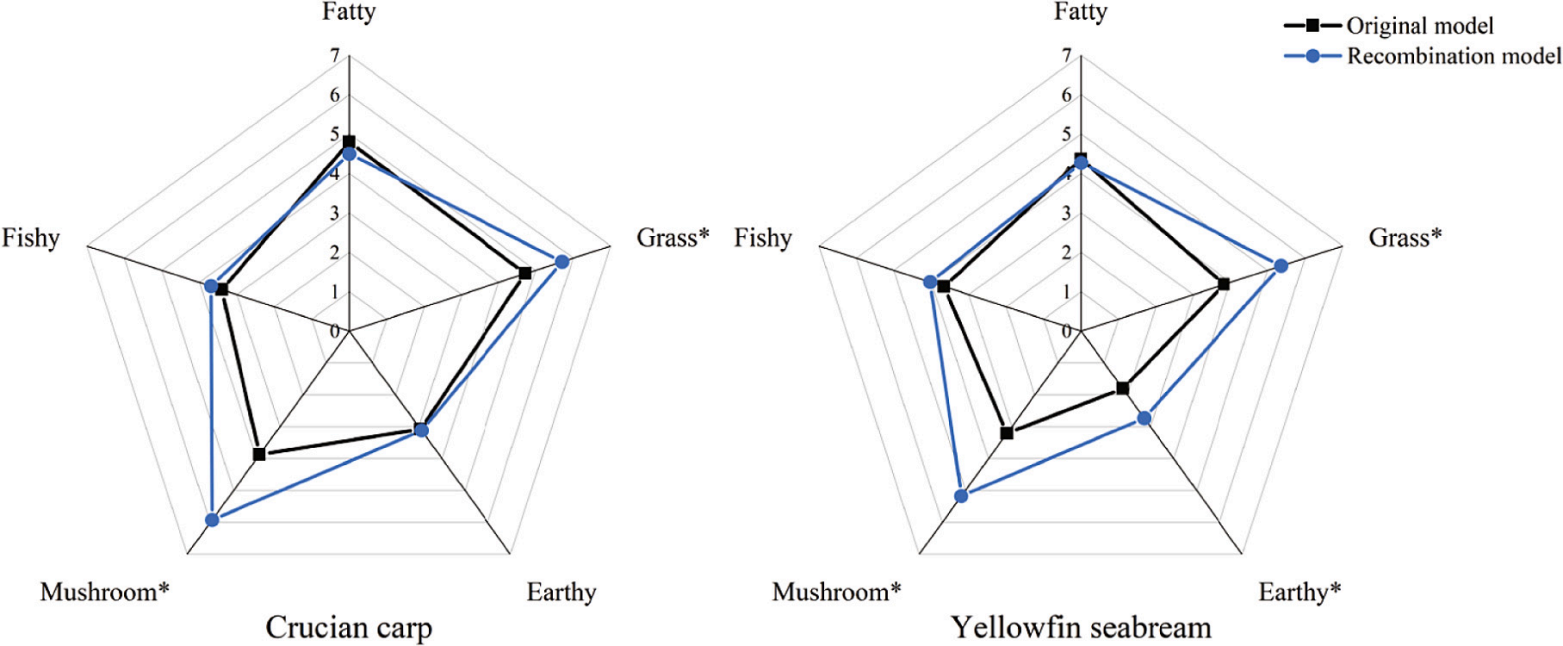
Aroma profiles of the original models and their recombination models for crucian carp and yellowfin seabream. (* represents *P* < 0.05).

Aroma recombination experiments were conducted using the volatile compounds with OAV ≥ 1 listed in Table 2 (see Table S3). The resulting recombined odor models for crucian carp and yellowfin seabream are shown in Fig. 4. Certain differences in sensory attributes were observed between the recombination models and the original fish samples, which may arise from interactions among odor compounds or the binding effects between the compounds and the matrix (Zhang, Yang, et al., 2022). In actual fish flesh, due to the embedding effect of muscle tissue and the hydrophobic binding of adipose tissue, volatile components are released continuously and gradually. In contrast, in the recombined samples, all the odor compounds were added at once without the constraint of the food matrix, leading to rapid release and resulting in higher overall odor intensity scores compared to the original samples. Additionally, the more complex composition of volatile compounds in real samples may exert masking effects, leading to reduced perceptual intensity of certain odor attributes (Wu et al., 2026). Although some odor attributes differed, no significant differences (*P* > 0.05) were found between the recombined and the original samples in the sensory evaluation of fishy odor. Furthermore, similarity analysis using the cosine similarity method revealed that the cosθ values between the recombined and the original models were close to 1 (0.984 for crucian carp; 0.981 for yellowfin seabream), indicating a high degree of similarity and confirming the success of the odor recombination system established in this study.

### 3.5 Omission analysis

Based on the combined results of OAV and GC-O analyses, volatile compounds with high OAV (≥10) and high odor intensity (≥2) were defined as key fishy odor-related components in this study (Table 4). The selection criteria (GC-O intensity ≥2 and OAV ≥ 10) were set to identify compounds with both clear sensory perception and high odor activity, thereby focusing the omission experiments on the most impactful odorants. Although (E,E)-2,4-dodecadienal had an OAV of 8, it was still included as a key odorant in crucian carp due to its relatively high odor intensity (2.75) and its status as a compound unique to this species.

**Table 4:** Effect of omitting key fishy odor components.

Fish species	Key fishy odor components	N	Significance of omission effect
Crucian carp	(E,Z)-2, 6-Nonadienal	9	**
	Nonanal	8	**
	(E,E)-2, 4-Nonadienal	8	**
	(E,E)-2, 4-Decadienal	8	**
	Hexanal	8	**
	1-Octen-3-ol	6	*
	(E,E)-2, 4-Dodecadienal	3	
Yellowfin seabream	(E,Z)-2, 6-Nonadienal	8	**
	Nonanal	8	**
	(E,E)-2, 4-Nonadienal	6	*
	(E,E)-2, 4-Decadienal	6	*
	1-Octen-3-ol	5	

Note: “N” represents the number of sensory evaluators (out of 10) who perceived a significant difference between the samples. * and ** indicate significant differences at *P* < 0.05 and *P* < 0.01, respectively.

**Fig. 5:**
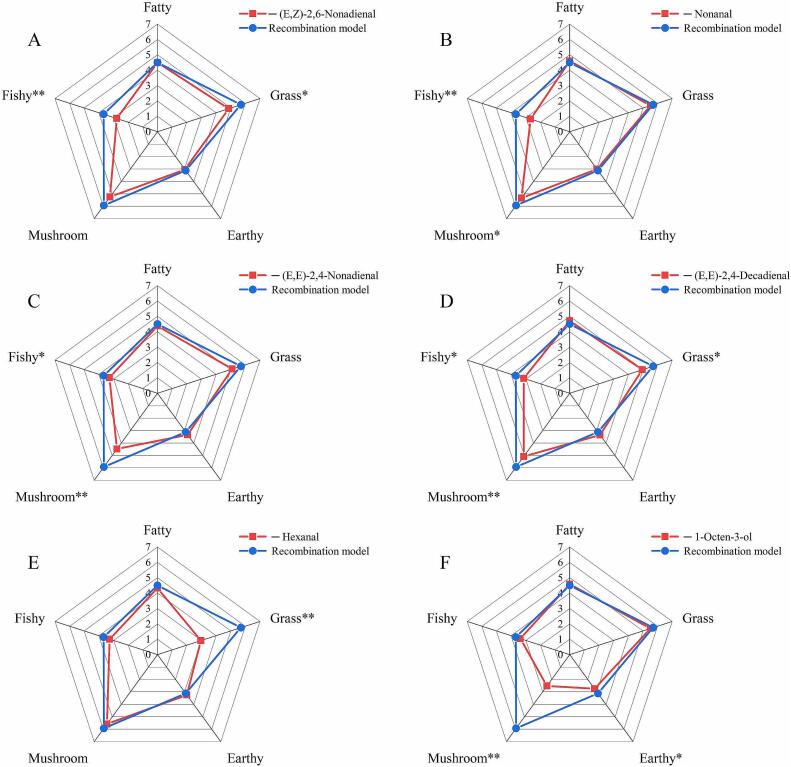
Aroma recombination and omission models of crucian carp. The blue and red lines represent the complete recombination model and the model omitting a key odorant, respectively. Significant differences from the complete model are indicated by * (*P* < 0.05) and ** (*P* < 0.01). (For interpretation of the references to colour in this figure legend, the reader is referred to the web version of this article.)

**Fig. 6:**
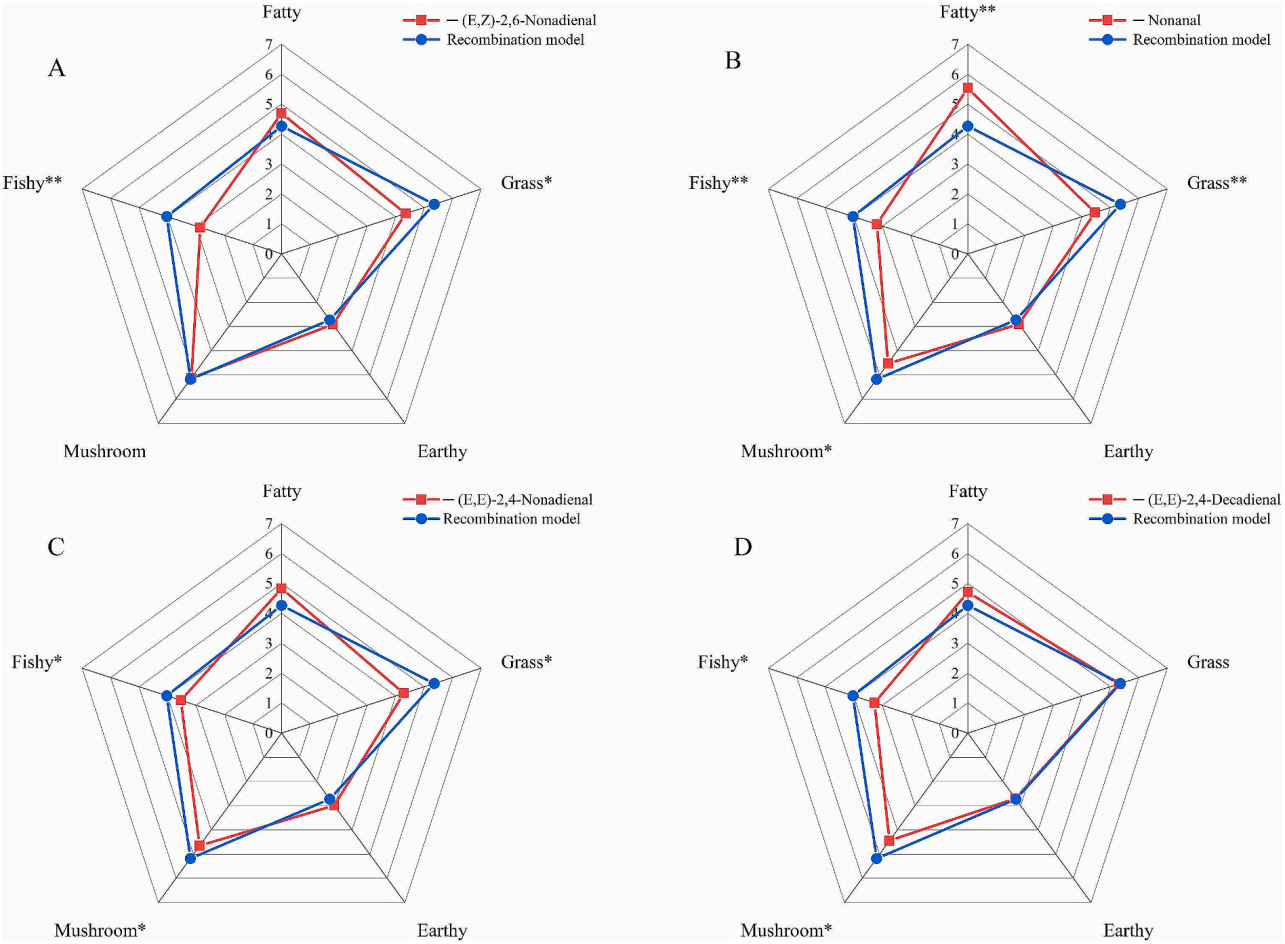
Aroma recombination and omission models of yellowfin seabream. The blue and red lines represent the complete recombination model and the model omitting a key odorant, respectively. Significant differences from the complete model are indicated by * (*P* < 0.05) and ** (*P* < 0.01). (For interpretation of the references to colour in this figure legend, the reader is referred to the web version of this article.)

Based on the recombined models, omission experiments were conducted on these key fishy odor-related components, and the impact of omitting individual components on the overall odor profile was evaluated through sensory analysis (Table 4). The omission of (E,E)-2, 4-dodecadienal in crucian carp and 1-octen-3-ol in yellowfin seabream did not significantly affect their respective recombination models. In contrast, the removal of the other key fishy odor components resulted in significant (*P* < 0.05) or highly significant (*P* < 0.01) effects on the recombined models. The specific impacts of omitting these compounds on various odor attributes are shown in Fig. 5, Fig. 6.

As shown in Fig. 5, the omission of (E, Z)-2,6-nonadienal (Fig. 5A) and nonanal (Fig. 5B) resulted in a highly significant reduction (*P* < 0.01) in the fishy odor intensity of crucian carp. The absence of (E,E)-2,4-nonadienal (Fig. 5C) and (E,E)-2,4-decadienal (Fig. 5D) also led to a significant decrease in the fishy odor intensity of crucian carp (*P* < 0.05). The removal of hexanal (Fig. 5E) caused a highly significant decrease (*P* < 0.01) in the grassy odor intensity of crucian carp. The omission of 1-octen-3-ol (Fig. 5F) significantly diminished the mushroom odor (*P* < 0.01) and earthy odor (*P* < 0.05) intensities in crucian carp. In this study, the OAVs of hexanal and 1-octen-3-ol in crucian carp reached 41 and 43, respectively. Furthermore, both hexanal and 1-octen-3-ol have been reported to significantly enhance the earthy odor of 2-MIB (Mahmoud & Buettner, 2017). This synergistic effect was also confirmed by Liu et al. (2017), who demonstrated that the presence of hexanal or 1-octen-3-ol significantly intensified the off-odor intensity caused by GSM and 2-MIB in channel catfish fillets. This synergistic interaction, combined with the exclusive presence of 2-MIB in crucian carp, contributes to its more complex and intense earthy-musty odor profile compared to yellowfin seabream. Therefore, the key fishy odorants in fresh crucian carp include (E,Z)-2, 6-nonadienal, nonanal, (E,E)-2, 4-nonadienal, (E,E)-2, 4-decadienal, hexanal, and 1-octen-3-ol.

As shown in Fig. 6, the omission of (E,Z)-2, 6-nonadienal (Fig. 6A) and nonanal (Fig. 6B) resulted in a highly significant reduction (*P* < 0.01) in the fishy odor intensity of yellowfin seabream. The absence of (E,E)-2, 4-nonadienal (Fig. 6C) and (E,E)-2, 4-decadienal (Fig. 6D) also led to a significant decrease (*P* < 0.05) in the fishy odor intensity of yellowfin seabream, indicating that these components are key fishy odorants in fresh yellowfin seabream. Furthermore, the removal of nonanal significantly diminished grassy (*P* < 0.01) and mushroom (*P* < 0.05) odors, while simultaneously causing a significant increase (*P* < 0.01) in the fatty odor score. This suggests that nonanal exerts a broad and profound influence on the overall odor profile of yellowfin seabream.

## 4 Discussion

The odor of fish is primarily composed of volatile compounds such as aldehydes, alcohols, and ketones. Among these, aldehydes typically have low odor thresholds and often impart fishy, grassy, and fatty notes. Alcohols generally contribute plant-like, aromatic, and earthy aromas (Zhang et al., 2023), with unsaturated alcohols having lower odor thresholds than their saturated counterparts and thus contributing more significantly to the overall odor profile. Ketones predominantly exhibit creamy and fruity aroma characteristics (Wang, Fan, et al., 2023). Fishy odor originates mainly from small-molecular aldehydes, alcohols, and ketones produced by the oxidative degradation of unsaturated fatty acids in fish tissue (Zhang et al., 2023). Compounds including hexanal, octanal, nonanal, (E)-2-octenal, (Z)-4-heptenal, (E,E)-2, 4-heptadienal, 1-octen-3-ol, and 2,3-octanedione have been reported as common contributors to fishy odors (An et al., 2020; Zhang et al., 2023). The interplay between flavor compounds and gut microbiota has recently emerged as a new frontier in food flavor research (Bhuiyan, 2026).

Currently, research on fish odor components in crucian carp is relatively limited. Cai et al. (2021) proposed ten compounds (OAV ≥ 1) as the main contributors to the overall odor of crucian carp, including 1-nonanol, 1-octen-3-ol, hexanal, decanal, octanal, and 1-nonanal. Using relative odor activity value (ROAV) analysis, which calculates the ratio of a compound's OAV to the maximum OAV in the sample (expressed as a percentage to rank its relative contribution to the overall odor profile), Wang et al. (2025) detected six key volatile organic compounds common to both the crucian carp meat and mucus: hexanal, nonanal, octanal, heptanal, 1-hexanol, and 1-octen-3-ol. Among these, hexanal and nonanal exhibited the highest ROAV values in both sample types, indicating their significant contribution to the fishy odor. By integrating GC–MS-O, OAV, and partial least squares regression (PLSR) analyses, Zhang et al. (2021) found that the fishy odor of crucian carp soup was significantly positively correlated with octanal, decanal, and 1-octen-3-ol, but significantly negatively correlated with (E,E)-2, 4-decadienal. In contrast, literature reports on odor components of yellowfin seabream are scarce. Zheng et al. (2025) used HS-SPME and GC–MS to compare differences in the volatile components of yellowfin seabream muscle under different salinity conditions, but did not analyze its fishy odor components.

OAV/ROAV analyses are commonly used to identify odor-active compounds, while GC–MS-O can directly determine the odor characteristics of individual components through sniffing. However, both methods are influenced by sample pretreatment techniques and are inadequate for evaluating interactions among components or the contribution of specific compounds to the overall odor profile (Zhang, Tang, et al., 2022). observed significant differences in the volatile compounds of fish soup when extracted using five different volatile extraction methods. In contrast to simply relying on OAV screening or GC-O sniffing to identify fishy compounds, this study systematically elucidated the composition and differences of fishy odor components in crucian carp and yellowfin seabream through a molecular sensory science approach. First, key odor-active compounds were screened via OAV analysis, and fishy odor components were identified through GC-O sniffing. On this basis, an aroma recombination model was constructed using components with OAV ≥ 1 to verify the effectiveness of volatile collection and the accuracy of qualitative/quantitative analysis. Subsequently, omission experiments were conducted to specifically evaluate the impact of compounds exhibiting high OAV (≥ 10) and high olfactory intensity (≥ 2.0) on the odor profile and fishy odor of the samples. This integrated strategy effectively combines the strengths of instrumental analysis and human sensory evaluation, providing the first systematic elucidation of key fishy odor-related components in crucian carp and yellowfin seabream.

Distinct differences exist in the flavor composition between freshwater and marine fish (Zhang et al., 2023). Compared to marine fish surimi (e.g., Pacific cod and Alaska pollock), freshwater crucian carp surimi contains a higher concentration of aldehydes, resulting in more pronounced characteristics, such as “river/fishy” and “grassy” notes (An et al., 2020). From a biological perspective, the differences in odor compounds identified in this study reflect distinct physiological metabolic and environmental adaptation pathways between the two fish species. The significantly higher levels of aldehydes in crucian carp primarily originate from lipid oxidation, likely related to its specific lipid composition and oxidative stability. Meanwhile, 2-MIB was detected only in crucian carp, which suggests its origin from microbial activity in freshwater environments. This compound is mainly produced by actinomycetes and cyanobacteria in freshwater and accumulates in fish through gill absorption (Zhou et al., 2024). In contrast, yellowfin seabream contained significantly higher (*P* < 0.05) concentrations of non-odor-active hydrocarbons. While hydrocarbons generally contribute little to fish odor due to their high odor thresholds, they can impart strong, irritating odors at high concentrations, potentially influencing the overall odor profile (Zhang, Cao, et al., 2019). The origin of hydrocarbons may stem from lipid auto-oxidation, carotenoid degradation, or dietary factors (Ma et al., 2020). Similarly, Selli and Cayhan (2009) reported relatively high levels of alkanes in the volatile profile of wild gilthead sea bream (*Sparus aurata*). Furthermore, Dinar and Picauly (2023) found that total hydrocarbon content was significantly higher in marine fish (Salmon, Tuna, and Mackerel) than in freshwater species (Tilapia, Catfish, and Trout), attributing this difference to habitat. Similarly, Bhuiyan et al., (2026) emphasized that marine contaminants including hydrocarbons are influenced by habitat-specific factors.These findings collectively indicate that the odor characteristics of fish are a comprehensive chemical expression resulting from the interaction of their specific growth environment, dietary sources, and intrinsic metabolic physiology. In particular, compared with yellowfin seabream, which contained higher concentrations of odor-inactive hydrocarbons, crucian carp exhibited higher levels of aldehydes, ketones, alcohols, and the earthy-musty compound 2-MIB, reflecting typical differences in volatile composition between freshwater and marine fish.

The limitations of this work include the following: (1) Not all odorants detected by GC-O could be successfully identified, particularly three unknown compounds that were described as fishy, which may affect the completeness of the final fishy odor profile, future studies using two-dimensional gas chromatography and/or more sensitive detection methods could help characterize such trace-level unknowns. (2) The study focused solely on the odorant composition of fresh fish muscle and did not investigate dynamic changes during storage or processing, which is an important direction for future research.

## 5 Conclusion

This study systematically characterized the volatile compound profiles and identified key fishy odorants in crucian carp and yellowfin seabream by combining HS-SPME, GC–MS-O, sensory evaluation, OAV analysis, aroma recombination, and omission experiments. The contents of aldehydes, alcohols, and ketones, were higher in crucian carp than in yellowfin seabream. The earthy, grassy, and mushroom odors were also significantly stronger in crucian carp*.* The fishy odor in both species resulted from the combined contribution of multiple volatile compounds. Key fishy odorants in crucian carp included (E,Z)-2, 6-nonadienal, nonanal, hexanal, (E,E)-2, 4-decadienal, (E,E)-2, 4-nonadienal, and 1-octen-3-ol, while yellowfin seabream contained (E,Z)-2, 6-nonadienal, nonanal, (E,E)-2, 4-decadienal, and (E,E)-2, 4-nonadienal. Notably, 2-MIB, a characteristic earthy-musty compound specific to freshwater fish was detected exclusively in fresh crucian carp. Together with the greater number and higher concentrations of key fishy odorants, crucian carp exhibited a more intense odor profile with a more complex composition of fishy odorants. These compositional differences provide a definitive chemical fingerprint that distinguishes the odor profile of freshwater crucian carp from that of marine yellowfin seabream.

Future studies should focus on how different storage conditions post-slaughter (e.g., temperature, time) and processing methods (e.g., frying, boiling) influence the composition and intensity of fishy odorants. Such research will provide a scientific foundation for optimizing the processing of fish-based products (e.g., fish soup) and controlling undesirable odors in aquatic foods.

## Ethical approval for animal experiments

All animal experimental procedures conducted in this study were reviewed and approved by the Institutional Animal Care and Use Committee (IACUC) of Jimei University (Xiamen, China; Permit Number: SCXK 2016–0006). All operations were strictly carried out in compliance with the institutional ethical standards and guidelines for the care and use of laboratory animals.

## Ethical approval for sensory evaluation

Informed consent was obtained from all trained panelists prior to their participation in the sensory study. The research protocol adhered to the ethical guidelines outlined in the Declaration of Helsinki.

## CRediT authorship contribution statement

**Yiqi Li:** Writing – original draft, Methodology, Investigation, Data curation, Conceptualization. **Shu Guo:** Writing – original draft, Formal analysis, Data curation, Conceptualization. **Chuanbo He:** Methodology, Investigation, Formal analysis. **Ruifang Wang:** Project administration, Methodology, Conceptualization. **Yuanhong Xie:** Methodology, Investigation, Conceptualization. **Hejian Xiong:** Writing – review & editing, Project administration, Methodology, Investigation, Funding acquisition, Data curation, Conceptualization. **Ying Ma:** Writing – review & editing, Validation, Supervision, Project administration, Methodology, Funding acquisition, Conceptualization.

## Declaration of competing interest

The authors declare that they have no known competing financial interests or personal relationships that could have appeared to influence the work reported in this paper.

## Data Availability

Data will be made available on request.
