# Supplementary material for: Analysis of key fishy odorants in crucian carp (Carassius auratus) and yellowfin seabream (Acanthopagrus latus)
Source: Food Chem X. 2026 Jul 29;38:104261. doi: 10.1016/j.fochx.2026.104261 (PMC13452400; doi:10.1016/j.fochx.2026.104261)
Supplement: Supplementary file 1 [file mmc1.docx]

Supplemental Table S1. List and sources of main chemical reagents

| Reagents Name | Manufacturer |
| --- | --- |
| Cyclohexanone | Sigma Aldrich Corporation, USA |
| N-alkanes (C8-C20) | Sigma Aldrich Corporation, USA |
| Hexyl alcohol | Sigma Aldrich Corporation, USA |
| Hexanal | Sigma Aldrich Corporation, USA |
| Heptanol | Sigma Aldrich Corporation, USA |
| Octanol | Sigma Aldrich Corporation, USA |
| 1-Octen-3-ol | Sigma Aldrich Corporation, USA |
| Octanal | Sigma Aldrich Corporation, USA |
| 3,5-Octadien-2-one | Sigma Aldrich Corporation, USA |
| (E,E)-2, 4-Heptadienal | Sigma Aldrich Corporation, USA |
| Nonanal | Sigma Aldrich Corporation, USA |
| Nonanol | Sigma Aldrich Corporation, USA |
| E-2-Octen-2-ol | Sigma Aldrich Corporation, USA |
| E-2-Octenal aldehyde | Sigma Aldrich Corporation, USA |
| (E,Z)-2, 6-Nonadienal | Sigma Aldrich Corporation, USA |
| (E,E)-2, 4-Nonadienal | Sigma Aldrich Corporation, USA |
| Heptaldehyde | Sigma Aldrich Corporation, USA |
| E-2-Nonenal | Sigma Aldrich Corporation, USA |
| Decanal | Sigma Aldrich Corporation, USA |
| E-2-Decenal | Sigma Aldrich Corporation, USA |
| Dodecanal | Sigma Aldrich Corporation, USA |
| (E,E)-2, 4-Dodecadienal | Sigma Aldrich Corporation, USA |
| Diisobutyl phthalate | Sigma Aldrich Corporation, USA |
| Caryophyllene | Sigma Aldrich Corporation, USA |
| E-2-Nonenal | Sigma Aldrich Corporation, USA |
| 2-Ethylhexanol | Sigma Aldrich Corporation, USA |
| Soil bromine | Sigma Aldrich Corporation, USA |
| 2-Methylisoborneol | Sigma Aldrich Corporation, USA |
| (E,E)-2,4-Dodecadienal | Sigma Aldrich Corporation, USA |
| Naphthalene | Sigma Aldrich Corporation, USA |
