# Supplementary material for: Analysis of key fishy odorants in crucian carp (Carassius auratus) and yellowfin seabream (Acanthopagrus latus)
Source: Food Chem X. 2026 Jul 29;38:104261. doi: 10.1016/j.fochx.2026.104261 (PMC13452400; doi:10.1016/j.fochx.2026.104261)
Supplement: Supplementary file 2 [file mmc2.docx]

Supplemental Table S2. Quantification calibration curves for volatile compounds in crucian carp and yellowfin seabream

| Compounds | Linear cquation | R^2^ | CI | Range (μg/L) | CF |
| --- | --- | --- | --- | --- | --- |
| Hexanal | Y=1.0236X - 0.02152 | 0.9921 | 44,56,72 | 5 - 700 | 7.83 |
| Hexanol | Y=3.0082X + 0.0032 | 0.9971 | 56,43,69 | 10 - 500 | 0.28 |
| Heptaldehyde | Y=5.9202X + 0.2110 | 0.9809 | 44,70 | 5 - 300 | 0.24 |
| Heptanol | Y=51.7347X - 1.7866 | 0.9954 | 70,41 | 15 - 150 | 0.03 |
| 2-Ethylhexanol | Y=76.7429X - 0.1346 | 0.9954 | 57,41,68 | 5 - 700 | 4.38 |
| 1-Octen-3-ol | Y=18.0238X - 1.5923 | 0.9884 | 57,43,72 | 10 - 500 | 0.02 |
| Octanal | Y=47.3623X-2.3180 | 0.9966 | 43,56,84 | 5 - 300 | 0.03 |
| (E,E)-2, 4-Heptadienal | Y=39.3344X - 1.1470 | 0.9989 | 81,53 | 15 - 150 | 0.03 |
| Nonanal | Y=7.0893X - 0.0353 | 0.9911 | 57,41,70 | 5 - 700 | 0.48 |
| (E)-2-Octen-1-ol | Y=31.5505X - 0.1352 | 0.9787 | 57,41 | 5 - 700 | 0.49 |
| E-2-Octenal aldehyde | Y=29.1307X - 0.0773 | 0.9970 | 41,55,70 | 5 - 700 | 0.53 |
| Octanol | Y=87.6208X - 0.3044 | 0.9862 | 56,41,84 | 5 - 700 | 1.56 |
| (E,Z)-2, 6-Nonadienal | Y=82.9692X - 0.1962 | 0.9987 | 41,70,69 | 5 - 700 | 1.50 |
| (E,E)-2, 4-Nonadienal | Y=166.813X - 1.2919 | 0.9939 | 81,41 | 5 - 700 | 0.36 |
| E-2-Nonenal | Y=89.1384X - 0.3402 | 0.9941 | 55,70,43 | 5 - 700 | 1.59 |
| Nonanol | Y=83.1691 - 0.4296 | 0.9957 | 56,55,70 | 5 - 700 | 4.72 |
| Decanal | Y=78.3512X - 0.2028 | 0.9829 | 57,43,41 | 5 - 700 | 1.38 |
| E-2-Decenal | Y=4.8312X - 0.0445 | 0.9935 | 70,83,55 | 5 - 700 | 0.37 |
| (E,E)-2, 4-Dodecadienal | Y=28.6135X - 0.2021 | 0.9911 | 81,41,67 | 5 - 700 | 0.34 |
| (E,E)-2, 4-Decadienal | Y=429.168X - 2.5144 | 0.9823 | 81,41,67 | 5 - 700 | 7.54 |
| Diisobutyl phthalate | Y=1.1661X + 0.0853 | 0.9904 | 149,57,41 | 15 - 150 | 0.003 |

Note: The correction factor (CF) was calculated using the following formula: CF=(A_S_/M_s_)/ (A_i_/M_i_);As where Aₛ represents the peak area of the quantitative ion (in SIM mode) of the internal standard (cyclohexanone), Aᵢ denotes the peak area of the quantitative ion (in SIM mode) of the standard chemical, Mₛ is the concentration of the internal standard (cyclohexanone), and Mᵢ is the concentration of the standard chemical.
