# Supplementary material for: Analysis of key fishy odorants in crucian carp (Carassius auratus) and yellowfin seabream (Acanthopagrus latus)
Source: Food Chem X. 2026 Jul 29;38:104261. doi: 10.1016/j.fochx.2026.104261 (PMC13452400; doi:10.1016/j.fochx.2026.104261)
Supplement: Supplementary file 3 [file mmc3.docx]

Supplemental Table S3. Volatile compounds for the reconstituted models of crucian carp and yellowfin seabream

| Compounds | Crucian carp | | Yellowfin seabream | |
| --- | --- | --- | --- | --- |
|  | Concentration (μg/kg) | OAV | Concentration (μg/kg) | OAV |
| (E,Z)-2, 6-Nonadienal | 1.2 | 267 | 2.57 | 571 |
| (E,E)-2, 4-Decadienal | 2.97 | 42 | 2.94 | 42 |
| Nonanal | 20.55 | 21 | 13.4 | 13 |
| (E,E)-2, 4-Nonadienal | 3.9 | 85 | 3.89 | 85 |
| 1-Octen-3-ol | 51.27 | 43 | 45.41 | 38 |
| Octanal | 27.01 | 8 | 27.44 | 8 |
| Hexanal | 185.66 | 41 | 100.88 | 22 |
| E-2-Nonenal | 2.02 | 20 | 1.97 | 20 |
| E-2-Decenal | 6.12 | 20 | 5.74 | 19 |
| Nonanol | 2.76 | 3 | 2.52 | 2 |
| Heptanol | 17.78 | 3 | 17.81 | 3 |
| (E,E)-2, 4-Heptadienal | 14.7 | 1 | 10.63 | <1 |
| Dodecanal | 1.63 | 3 | 3.45 | 7 |
| 2,4-Dodecanedional | 4.1 | 8 | ND | ND |
| Decanal | 1.41 | 14 | 1.47 | 15 |
| Heptaldehyde | ND | ND | 20.53 | 7 |
| 3,5-Octadien-2-one | ND | ND | 2.51 | 3 |
| E-2-Octene-1-ol | 4.06 | <1 | ND | ND |

Note: Aroma recombination was performed using odor compounds with OAV ≥ 1 listed in Table 2. (Z,Z)-3,6-nonadienal and 2,3-octanedione (OAV > 1) were not included due to the unavailability of commercial standards, while E-2-octen-1-ol (OAV < 1) was included for its potential contribution to fishy odor. “ND” denotes “not detected” or “not added”.
